# Optimization of temperature and parental effects on seed germination and seedling growth in pistachio varieties

**DOI:** 10.3389/fpls.2026.1844174

**Published:** 2026-08-05

**Authors:** Adela Hyseni, Carlos Trapero, María Lovera, Pedro Valverde

**Affiliations:** 1 Departamento de Agronomía, ETSIAM, Universidad de Córdoba, Córdoba, Spain; 2 Departamento de Fruticultura Mediterránea, IFAPA, Córdoba, Spain

**Keywords:** early-stage selection, germination percentage, pistachio breeding, *Pistacia vera* L., radicle growth, seed physiology, seedling vigor, temperature effect

## Abstract

**Introduction:**

Pistachio (*Pistacia vera* L.) is a temperate and semi-arid tree crop of growing economic importance worldwide, yet new cultivar development is needed to enhance adaptation to diverse growing environments. Optimizing seed germination and early seedling growth is essential for accelerating breeding programs in long-generation tree crops such as pistachio. This study aimed to (i) determine the optimal temperature for seed germination and (ii) evaluate the effects of female parent, field origin, and seed weight on germination, seed emergence and seedling performance.

**Methods:**

Seed from open-pollinated pistachio cultivars were collected from experimental orchards in southern Spain and subjected to controlled germination conditions. Five temperature regimes (5, 20, 24, 28, and 32 °C) were tested, and germination percentage, germination velocity index (GVI) and radicle growth were monitored over time.

**Results and discussion:**

Temperature was the main factor influencing germination and radicle growth. No germination occurred at 5 °C, whereas the highest germination percentage and maximum radicle growth were recorded at 28 °C. Temperatures between 20 °C and 32 °C showed similar final germination rates, although early germination was faster at higher temperatures. Female parent significantly influenced seedling growth but had limited effects on emergence percentage. ‘Kalehghouchi’, ‘Lost Hills’ and ‘Kastel’ exhibited superior seedling vigor, while ‘Larnaka’ and ‘Joley’ showed lower performance. Field origin had a minor effect on germination but significantly affected later seedling growth, suggesting that seed origin, through a combination of environmental and genetic factors, may influence seed physiological quality. Seed weight differed among cultivars and was positively correlated with seedling growth, but not with germination percentage. Overall, results demonstrate that germination in *P. vera* can be efficiently achieved without complex dormancy-breaking treatments under optimal temperature conditions. The identification of key factors affecting early seedling performance provides practical guidelines for improving seedling production and selection criteria to accelerate pistachio breeding programs.

## 1 Introduction

The genus *Pistacia*, which belongs to the Anacardiaceae family, contains more than 11 tree and shrub species (Zohary, 1952). Among them, *Pistacia vera* L. is the most commercially important due to its large edible nuts, making it the key species in the genus (Ferguson et al., 2005). Global pistachio production reached approximately 1.3 million tons in 2023, with USA, Iran, and Türkiye leading the production, accounting for the 90% of the global share (FAO, 2023). In Spain, pistachios are currently grown on around 85,845 ha with a total production of 24,039 t, making the country the 5^th^ largest producer worldwide. Forecasts indicate an increase in the coming years, with only around 33% of the existing area in full production and an annual increase of over 6,008 hectares (MAPA, 2025).

In Spain, pistachio cultivation is currently dominated by a limited number of commercial varieties, represented by ‘Kerman’, accounting for 52% of the agricultural area in 2020. Followed by ‘Sirora’ with 6% of the area, and others such as ‘Larnaka’, ‘Golden Hills’, ‘Lost Hills’ and ‘Aegina’, none of which exceed 2% of the cultivated area (MAPA, 2024). The limited number of dominant varieties in Spain, particularly the reliance on ‘Kerman’ and ‘Sirora’, makes the sector vulnerable to pests, diseases, and climate change challenges. Therefore, the successful introduction of new, well adapted, and high yielding cultivars would be immensely beneficial, offering greater genetic diversity to the Spanish pistachio industry and helping to secure its long-term viability.

To achieve this necessary diversification, genetic enhancement of pistachio is critical for developing superior female and male cultivars, as well as improved rootstock (Kallsen et al., 2009). Although pistachio has been less genetically studied compared to other major fruit trees, its expansion suggests a promising potential for breeding efforts. Most dedicated programs have been carried out by the University of California pistachio cultivar improvement program, initiated in 1989, which have provided valuable information on the genetic control of agronomical and phenological traits (Chao et al., 2003) and have resulted in the release of new competitive cultivars (Parfitt et al., 2007). Dedicated rootstock programs in California have also focused on developing disease resistant genotypes (Ashworth, 1984; Ferguson and Haviland, 2016). Although other programs were active in Catalonia (Spain) and Turkey, they have since been discontinued (Sheikhi et al., 2019). A major limitation of these traditional programs is the long generation time, often exceeding 15 years from initial cross to cultivar release. Pistachio worldwide, including Spain, now faces unprecedented challenges that necessitate the development of new varieties through advanced plant breeding techniques capable of significantly shortening these lengthy breeding cycles. Given that pistachio is a long-generation tree crop, breeding cycles traditionally require 8–10 years to complete a single generation (from making crosses to obtaining progeny plants for evaluation). However, this can be shortened to a 4–6 year cycle by growing seedlings under ideal conditions without nutrient and water limitations (Badenes and Byrne, 2012). Since most traits exhibit sufficient heritability, indicating promising potential for selecting superior genotypes (Chao et al., 1997), the strategy must focus on accelerating all early stages of the breeding process.

To generate the sexually propagated plants needed for genetic improvement, the mass production of new pistachio genotypes relies on propagation by seed (Ferguson and Haviland, 2016). Genotypes with the best characteristics are selected and then grafted onto rootstocks to establish productive orchards. Therefore, an important requirement for accelerating the breeding process is the optimization of *P. vera* germination and breeding protocols to successfully generate diverse and viable sexually propagated plants. Optimizing each parameter involved in seed germination is essential to reduce the time required for developing new varieties and to improve overall breeding efficiency.

Seed germination is a fundamental stage in plant life cycle and a crucial determinant of seedling establishment and crop success. Temperature is a critical factor influencing seed germination, as it affects both germination percentage and rate (Bewley and Black, 1994), and regulates numerous physiological and biochemical processes such as enzyme activation, hormonal regulation and cell elongation (Willis et al., 2014). In the genus *Pistacia*, temperature responses vary by species: *P. lentiscus* germinates optimally between 20 °C-25 °C, while *P. atlantica* performs best around 25 °C (Lefi et al., 2023). However, temperature alone is often insufficient to ensure successful germination, especially due to seed dormancy. Dormancy, primarily caused by the hard sclerotic endocarp that limits embryo expansion, is a significant barrier in *Pistacia* species (Bewley and Black, 1994; İsfendiyaroğlu and Özeker, 2001). In order to optimize and accelerate the seed germination process mechanical scarification and cold treatment are needed (Lefi et al., 2023). For instance, *P. khinjuk* responded positively to dormancy breaking treatments, which also improved seedling development (Acar et al., 2017).

In *P. vera*, pretreatment with water soaking is shown to be beneficial. Water soaking for 36–48 hours has shown significant increases in germination and enhanced seedling growth (Abou Rayya et al., 2018; Köksal et al., 1994), while longer durations can sometimes reduce vigor (Esmaeilpour and Van Damme, 2014). The strong correlation between water uptake and germination success emphasizes the influence of soaking time and solution composition (Bashabsheh et al., 2018). Furthermore, the use of gibberellic acid (GA_3_) has been effective in shortening germination time and promoting early seedling growth (Ak et al., 1994).

Seed size also plays a direct role in germination success, impacting germination time, percentage, and seedling vigor (Souza and Fagundes, 2014; Murali, 1997; van Mölken et al., 2005; Yang et al., 2020). In cashew (*Anacardium occidentale*), medium-weight seeds showed superior germination characteristics compared to other classes. However, seed weight did not influence seedling vigor, indicating that initial seed mass primarily affects germination performance rather than subsequent seedling development (Oyekale et al., 2019). Similarly, in peaches, heavier seeds were associated with a higher germination percentage and faster germination (Malcolm et al., 2003).

Given the long juvenile period of pistachio and the limited research on germination optimization, improving early developmental stages is essential to accelerate breeding programs. Temperature is a key factor regulating germination processes, however its optimal range for *P. vera* remains insufficiently defined under controlled conditions. In addition, the influence of maternal, field conditions and seed weight on germination and early seedling growth is not fully understood. A clearer understanding of these factors could improve seedling production efficiency and facilitate early-stage selection in pistachio breeding programs. Therefore, the objectives of this study were to: (i) determine the optimal temperature for pistachio seed germination percentage and germination velocity index (GVI) (ii) evaluate the effects of female parent, field origin, and seed weight on seed emergence, germination, and seedling growth.

## 2 Material and methods

### 2.1 Experimental fields and fruit sampling

In 2023 and 2024, during the pistachio harvesting season in Spain (September/October), 1 kg of pistachio fruits were harvested per block and cultivar in three experimental fields in the south of Spain: ‘Guadix’ (Granada province), ‘Chirivel’ (Almeria province) and ‘Carmona’ (Seville province). Samples were collected from 11 pistachio cultivars by open-pollination (‘Lost Hills’, ‘Golden Hills’, ‘Kalehghouchi’, ‘Mateur’, ‘Sirora’, ‘Kastel’, ‘Aegina’, ‘Avdat’, ‘Kerman’, ‘Larnaka’ and ‘Joley’), although some cultivars were only present in the ‘Guadix’ field and thus were not sampled in ‘Chirivel’ or ‘Carmona’. Pistachio fruits were randomly harvested by hand once they reached maturity. The day after the collection, the hull (exocarp) was removed in the laboratory, and the fruits were air-dried at room temperature for several days. Once dry, the shell (endocarp) was removed. The final samples of fruit were randomly selected for each subsequent experiment.

‘Guadix’ experimental field was planted in 2012 on a 1.58 ha surface. It has an experimental design of 4 randomized blocks, 11 varieties per block, 4 trees per row, with a tree spacing of 7 × 6 m at latitude 37°16’37.6”N and longitude 3°03’57.8”W. The maximum, average, and minimum temperatures from 2022 to 2024 are 21.3 °C, 13.7 °C, and 6.4 °C respectively. The average annual precipitation was 197.4 mm in 2022, 102.8 mm in 2023, and 85.4 mm in 2024 (IFAPA, 2024).

The second field ‘Chirivel’ was planted in 2019 with a 1.3 ha surface. It has 4 randomized blocks, 11 varieties per block, 4 trees per row, with tree spacing of 7 × 7 at a latitude 37°35’45.7”N, 2°17’21.6”W. The maximum, average, and minimum temperatures from 2022 to 2024 are 20.8 °C, 13.9 °C, and 7.6 °C respectively. The average annual precipitation was 151.5, 100.4 and 69.4 mm in 2022, 2023 and 2024 respectively (IFAPA, 2024).

The third field located in ‘Carmona’ was planted in 2017 with a surface of 0.6 ha and tree spacing of 7 x 5 m. It is located at latitude 37°24’18.9”N and longitude 5°28’19.7”W in Sevilla province. The maximum, average, and minimum temperatures from 2022 to 2024 are 27 °C, 18.7 °C, and 10.7 °C respectively. The average precipitation of 2022, 2023 and 2024 was 155.8, 167.2 and 258.4 mm respectively (IFAPA, 2024).

### 2.2 Temperature effects on seed germination and seedling growth

To evaluate the effect of temperature on seed germination, seeds from three open-pollinated pistachio cultivars (‘Kerman’, ‘Mateur’ and ‘Lost Hills’) were harvested from the ‘Carmona’ (Seville) experimental field.

Prior to sowing, the seeds were soaked in water for 24 h and subsequently disinfected for 24 h (5 mL/L of a 50% thiram solution) making a total of 48 h of hydration (Köksal et al., 1994).

Seeds were sown in perforated trays (33 × 22.5 cm) containing moistened perlite and incubated in darkness in controlled-environment chambers under five constant temperature regimes (5, 20, 24, 28, and 32 °C) and a constant relative humidity of approximately 80% monitored via the chamber’s digital display. The temperatures from 20 °C to 32 °C were selected to cover the range of warm conditions likely to support germination in P. vera, while the 5 °C treatment was included to establish the lower thermal limit for germination under constant incubation conditions. The experiment followed a randomized complete block design with three replicates trays per treatment, each consisting of 16 seeds per cultivar. Seeds were randomly collected from multiple trees within each block to obtain representative samples of each cultivar. Each tray was considered the experimental unit.

Germination was first recorded two days after sowing, corresponding to five days after the start of hydration (D5), and subsequently assessed every two days until day 13 (D13), where germination stabilized and no further significant increases were observed. Germination was considered successful when the radicle reached a length of 4 mm (Ardakani, 2015). Seeds were evaluated for both germination and radicle length at each assessment time. Radicle length (mm) of each pistachio seed was measured using a digital caliper. To quantify germination speed, the Germination Velocity Index (GVI) was calculated for each experimental unit as GVI = Σ (new germinations on day i/day i), following Maguire (1962), where new germinations represent the difference in cumulative germination percentage between consecutive measurement days.

### 2.3 Female parent effects on seed emergence and seedling growth

To evaluate the influence of different female parents, seeds were obtained via open pollination from 11 female pistachio varieties cultivated at the ‘Guadix’ experimental field: ‘Lost Hills’, ‘Golden Hills’, ‘Kalehghouchi’, ‘Mateur’, ‘Sirora’, ‘Kastel’, ‘Aegina’, ‘Avdat’, ‘Kerman’, ‘Larnaka’ and ‘Joley’ all selected from the ‘Guadix’ experimental field. The process of germination was the same as indicated in section 2.2. The germinated seeds were transplanted into 5 L pots containing a mix of 40% coconut fiber, 30% blond peat moss, 15% black peat moss and 15% perlite, and placed in a glasshouse at a day/night temperature regime of 28/20 °C (± 2 °C) with natural daylight.

After sowing, emergence percentage and seedling height was evaluated. Seedling height (cm) was measured from the stem base to the apical leaf three weeks after sowing (H1), and a second measurement was taken three weeks later (H2). The experiment consisted of 4 randomized blocks, each containing 30 seeds, and was conducted during the 2023 and 2024 harvesting seasons.

### 2.4 Field site effects on seed emergence and seedling growth

To assess the field effect on emergence percentage and seedling growth, seeds from two open-pollinated female varieties ‘Kerman’ and ‘Lost Hills’ were collected. Seeds were harvested from three experimental fields: ‘Guadix’, ‘Chirivel’ and ‘Carmona’ (described in 2.1) over two study years (2023 and 2024). The experiment followed a randomized block design with 4 blocks per field and approximately 30 seeds per block for each cultivar and field. Emergence percentage and seedling growth were evaluated as described above. The study was conducted in 2023 and 2024 under the same experimental conditions.

### 2.5 Seed weight effects on seed germination and seedling growth

The effect of seed weight was evaluated in 11 female pistachio varieties (‘Aegina’, ‘Avdat’, ‘Joley’, ‘Kalehghouchi’, ‘Kastel’, ‘Larnaka’, ‘Mateur’, ‘Sirora’, ‘Golden Hills’, ‘Kerman’ and ‘Lost Hills’) in open pollination grown in the ‘Guadix’ experimental field. After harvest, the seeds were air-dried at controlled room temperature for several days. Subsequently, the shells were carefully removed, and seed weight was determined for each cultivar using a precision balance. Subsequently the seeds were put to germinate, sown and then taken to nursery conditions. Germination percentage and seedling growth parameters were recorded according to the procedures described in sections 2.2 and 2.3. The experimental design was structured into 4 randomized blocks, one tree per block, and 25 seeds per tree. This experiment was conducted during the 2023 harvesting season.

### 2.6 Statistical analysis

The statistical analyses were performed using Statistix 10.0 program (Analytical Software, Tallahassee, FL, USA). Graphs were generated using GraphPad Prism version 8. Prior to analysis, data were tested for homogeneity of variances. For the temperature experiment, germination percentage and radicle length were analyzed using a one-way analysis of variance (ANOVA). Cultivar effects and cultivar × temperature interactions were tested, and as no significant interactions were detected, data were averaged across cultivars. To evaluate temperature effects over time, germination percentage was analyzed separately for each day of measurement, and temperature means were compared within each day using the Tukey HSD test at *P* ≤ 0.05. Radicle length was analyzed using two-way ANOVA with temperature and day of measurement as fixed factors. Mean separation was performed using Tukey HSD tests when significant effects were detected. To quantify germination speed, the Germination Velocity Index (GVI) was calculated for each experimental unit as GVI = Σ (new germinations on day i/day i), following Maguire (1962), where new germinations represent the difference in cumulative germination percentage between consecutive measurement days. To assess female parent effects on emergence percentage and seedling height, one-way ANOVA was performed across the 11 female pistachio varieties. Data from two growing seasons (2023 and 2024) were included in the analysis. Field site effects on emergence percentage and seedling height were evaluated using factorial ANOVA, with field location, variety, and their interaction as fixed factors. Seed weight differences among varieties were analyzed by one-way ANOVA. Relationships between seed weight, germination percentage, and seedling growth were assessed using Spearman correlation coefficients.

## 3 Results

### 3.1 Temperature effects on seed germination and seedling growth

Cultivar had no significant effect on seed germination and radicle length (*P* = 0.83 and *P* = 0.12 respectively), whereas temperature significantly affected both parameters (*P* < 0.0001 and *P* < 0.05 respectively). Variety × temperature interaction was not significant (*P* > 0.05).

**Figure 1:**
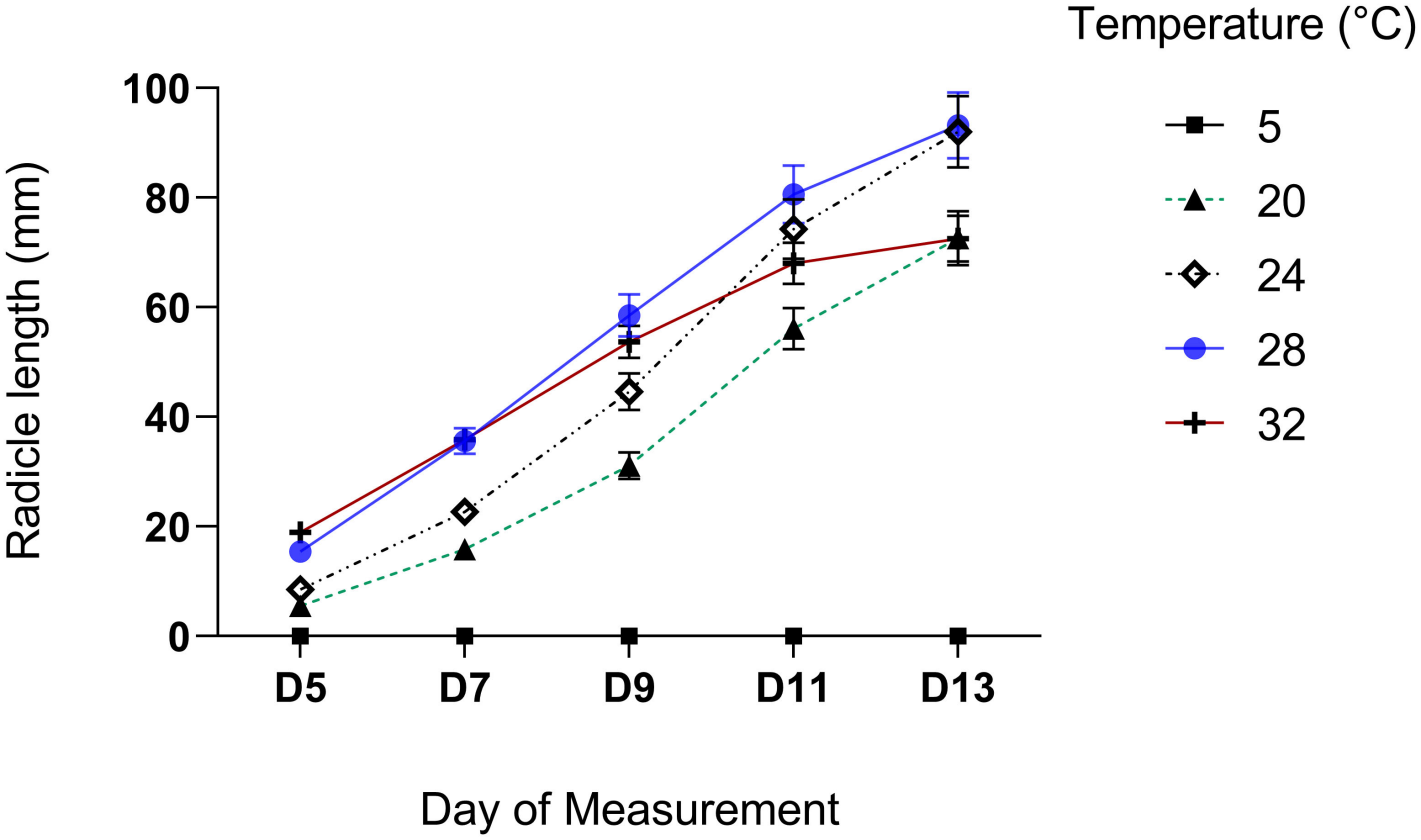
Evolution of radicle length of pistachio seeds at five temperature treatments measured on five non-consecutive days (every two days). Values represent the mean of ‘Kerman’,’Mateur’, and ‘Lost Hills’ × open pollination. Vertical bars indicate the standard error.

As shown in **Figure 1**, radicle emergence had its initial growth observed at 20 °C on D5, while no emergence was recorded at 5 °C throughout the experiment. Radicle growth increased progressively with temperature from D5 to D9, with higher mean lengths at 24-32 °C compared to 20 °C. By D9, radicle length reached 31.1 mm at 20 °C, 44.6 mm at 24 °C, 58.5 mm at 28 °C, and 53.7 mm at 32 °C. Maximum growth was consistently observed at 28 °C, peaking at 80.3 mm on D11 and 93.1 mm on D13, followed closely by 24 °C, whereas growth at 32 °C declined at later stages, likely due to increased fungal development.

Germination percentage of pistachio seeds varied significantly with temperature. Overall, the highest mean germination was recorded at 28 °C (91.7%), followed closely by 20 °C (91.0%), while slightly lower values were observed at 32 °C (88.9%) and 24 °C (88.2%), no germination occurred at 5 °C throughout the experiment. On the first evaluation date (D5), seeds incubated at 32 °C showed significantly higher germination compared to 20 °C, while 24 °C and 28 °C showed intermediate values, not significantly different from either 20 °C or 32 °C. From D7 onward, germination percentages among 20 °C, 24 °C, 28 °C, and 32 °C did not differ significantly within each measurement day (**Table 1**).

**Table 1:** Mean germination percentages of pistachio seeds measured on five sampling days at five different temperatures.

Day of measurement	Temperature				
	5 °C	20 °C	24 °C	28 °C	32 °C
D5	0 c	56.9 b	68.0 ab	76.3 ab	81.2 a
D7	0 b	79.8 a	79.8 a	86.1 a	84.7 a
D9	0 b	86.1 a	84.0 a	88.8 a	87.5 a
D11	0 b	90.2 a	86.8 a	91.6 a	88.8 a
D13	0 b	90.9 a	88.1 a	91.6 a	88.8 a

*Germination values followed by the same letter are not significantly different according to Tukey HSD test (*P* = 0.05). Letter groupings indicate comparisons among temperatures within the same day. Values represent means across three varieties.

The Germination Velocity Index (GVI) confirmed that germination speed was significantly affected by temperature (*P* < 0.0001). No germination was recorded at 5 °C (GVI = 0.00), while temperatures from 20 °C to 32 °C showed similar GVI values with no significant differences among them (Tukey HSD, P > 0.05). However, 28 °C recorded the numerically highest GVI (17.21), followed closely by 32 °C (17.17), 24 °C (16.09) and 20 °C (15.87), consistent with the faster early germination and superior radicle growth observed at 28 °C throughout the experiment.

### 3.2 Female parent effects on seed emergence and seedling growth

**Figure 2:**
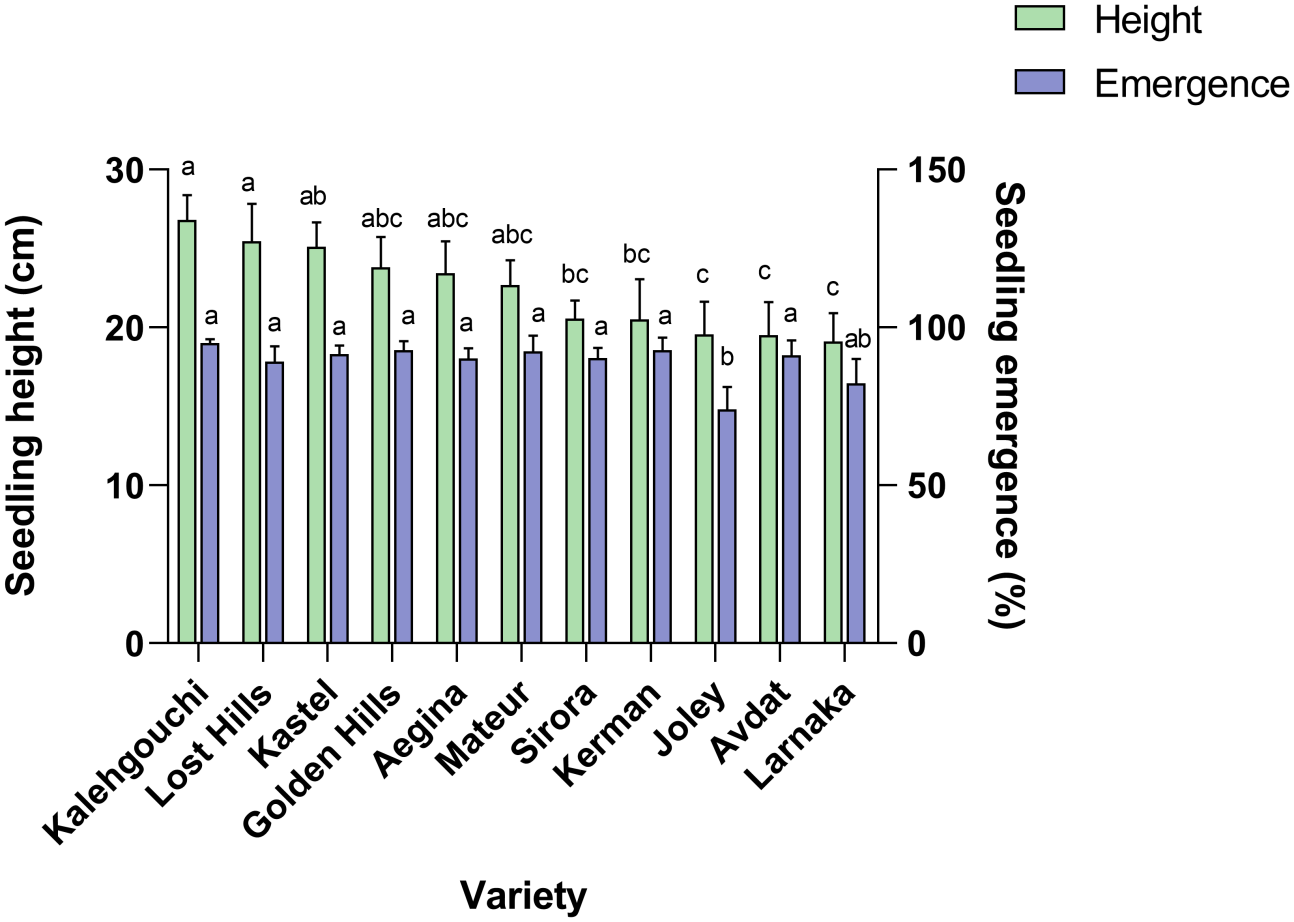
Mean seedling height (cm) and emergence (%) of 11 female varieties from ‘Guadix’ field, in two years of study (2023 and 2024). Vertical bars in the columns indicate the standard error. Values followed by the same letter are not significantly different according to LSD testing at *P* = 0.05.

Although for most cases there were no significant differences in emergence percentage among cultivars tested in the two years of study, ‘Kalehghouchi’ exhibited the highest mean emergence percentage (95%), followed by ‘Kerman’ and ‘Golden Hills’ both (92.8%). In contrast, ‘Larnaka’ and ‘Joley’ recorded the lowest emergence percentage, with 82.2% and 74%, respectively (**Figure 2**).

Regarding seedling growth, significant differences were observed between varieties.

‘Kalehghouchi’ showed the highest mean value (26.8 cm), followed by ‘Lost Hills’ (25.4 cm), and ‘Kastel’ (25.1 cm). Conversely, the varieties with the lowest average vigor were ‘Joley’ (19.5 cm), ‘Avdat’ (19.5 cm), and ‘Larnaka’ (19.1 cm), which corresponded to the lowest observed growth rate.

### 3.3 Field site effects on seed emergence and seedling growth

**Figure 3:**
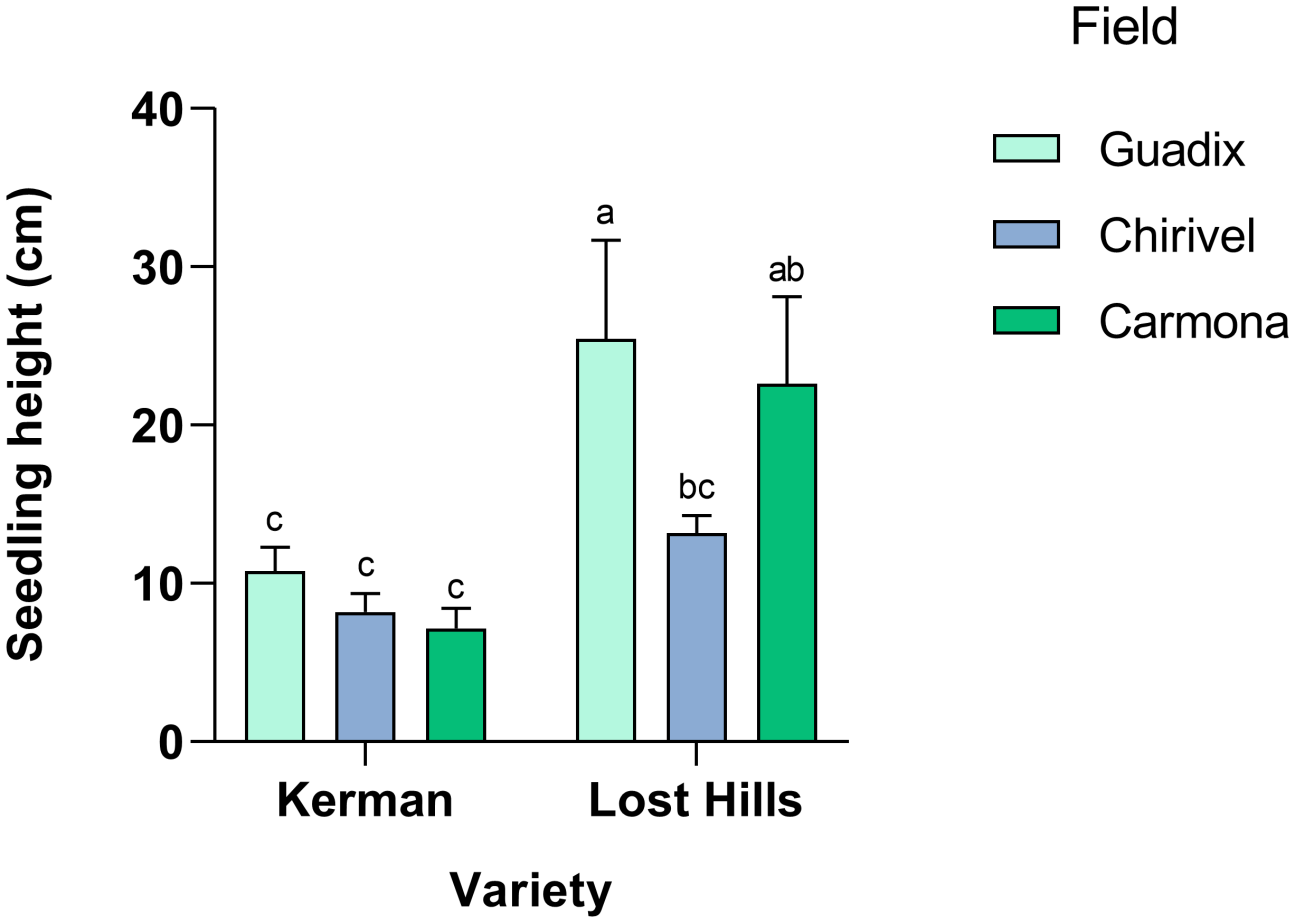
Seedling height (H1) (cm) measured three weeks after sowing, of two varieties ‘Kerman’ and ‘Lost Hills’ from three fields (‘Guadix’, ‘Chirivel’, ‘Carmona’) in two years of study (2023 and 2024). Vertical bars in the columns indicate the standard error. Values in each column followed by the same letter are not significantly different according to LSD testing at *P* = 0.05.

‘Lost Hills’ seedlings exhibited greater height compared to seedlings from ‘Kerman’, which displayed relatively uniform growth across all three fields. ‘Lost Hills’ exhibited a significantly higher percentage of emergence (96.27%) compared to ‘Kerman’ (78.37%). In particular, ‘Lost Hills’ reached 100% emergence from ‘Carmona’ field, while ‘Kerman’ seeds from the same field showed the lowest (68.68%) (**Figure 3**).

Field site significantly affected the second seedling height measured (*P* = 0.04) while the varietal effect was not significant (*P* = 0.42). The interaction between variety and field was not significant for either emergence or seedling height (*P* > 0.05).

A significant effect of female variety on early seedling height (H1) was revealed (*P* < 0.05), whereas field site and the field × variety interaction were not significant. In contrast, later seedling height (H2) was significantly affected by field site (*P* < 0.05), while female variety and the interaction term were not significant. Emergence percentage was significantly influenced by female variety (*P* = 0.008), with no significant effects of field site or interaction.

### 3.4 Seed weight effect on seed germination and seedling growth

**Figure 4:**
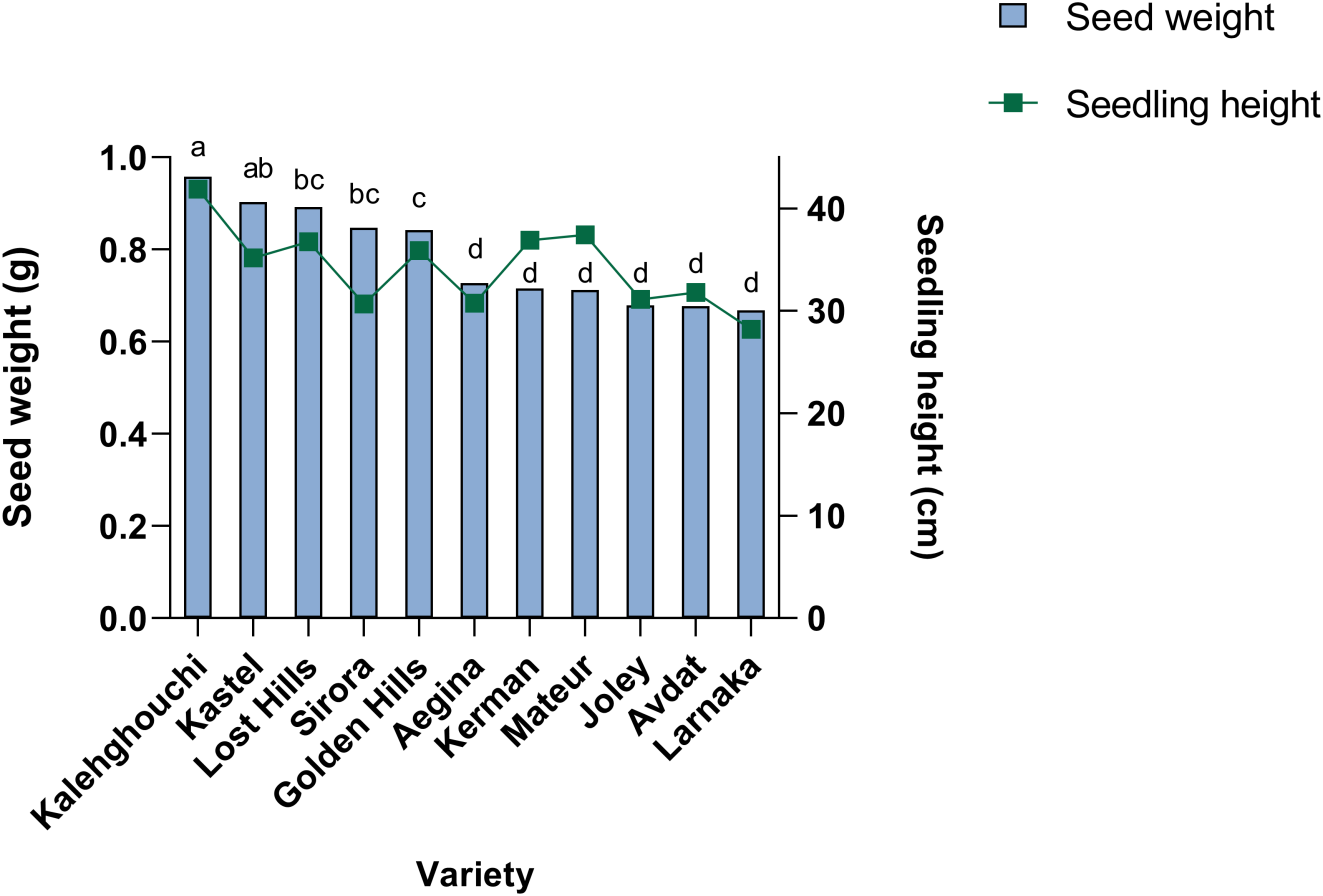
Seed weight (g) and height (cm) measured six weeks after sowing of 11 female varieties from ‘Guadix’ field, in two years of study (2023 and 2024). Vertical bars in the columns indicate the standard error. Values in each column followed by the same letter are not significantly different according to LSD testing at *P* = 0.05.

Seed weight differed significantly among the pistachio varieties (*P* < 0.05). ‘Kalehghouchi’ showed a higher seed weight with a mean of 0.95 g, followed by ‘Kastel’ with a mean of 0.9 g. ‘Lost Hills’, ‘Golden Hills’ and ‘Sirora’ showed intermediate seed weight (0.89 g, 0.84 g and 0.84 g respectively). ‘Joley’, ‘Avdat’, and ‘Larnaka’ had the lowest seed weight (0.67, 0.67 and 0.66 respectively) (**Figure 4**).

According to the Spearman correlation analysis, seed weight and seedling growth had a moderate significant correlation (r = 0.46), whereas a weak and no significant correlation was found for seed weight and germination (r = 0.07).

## 4 Discussion

Optimizing germination, seedling vigor, and parental selection is fundamental for improving the breeding cycle in perennial crops such as pistachio. Evidence from other woody crops indicates that early seedling growth can be linked to later performance and a shorter juvenile phase, highlighting the relevance of these traits for early selection (Rallo et al., 2008). However, information on these developmental stages in *Pistacia vera* L. remains limited. Strengthening knowledge of the factors influencing germination and initial seedling development is therefore crucial for enhancing breeding efficiency and seedling production. In this study, we addressed two key aspects: identifying the optimal temperature for pistachio seed germination and evaluating the parental and environmental effects on germination and early seedling growth across different female varieties.

### 4.1 Temperature effects on seed germination and seedling growth

In our study, temperature was identified as the most critical factor influencing both pistachio seed germination and seedling growth. This high influence confirms earlier studies on other crops, emphasizing temperature as a decisive factor in regulating physiological and biochemical processes during germination (Bewley, 1997; Willis et al., 2014).

Final germination percentage was similar across temperatures from 20 °C to 32 °C, with no statistically significant differences detected from D7 onwards. However, early germination speed differed among temperatures, with seeds incubated at 28 °C and 32 °C showing significantly higher germination percentages at D5 compared to 20 °C. The temperature of 28 °C was identified as most favorable for radicle development, producing the greatest radicle length throughout the experiment. These findings align with Lefi et al. (2023), who reported similar optimal temperature ranges for *Pistacia* species, although specific temperature optima can vary between species.

The Germination Velocity Index (GVI) further supported the finding that temperatures between 20 °C and 32 °C produced comparable germination speed, with no statistically significant differences among them. Nevertheless, 28 °C recorded the numerically highest GVI (17.21), consistent with the superior radicle growth observed at this temperature throughout the experiment. These results suggest that while a broad range of warm temperatures is suitable for pistachio germination, 28 °C represents the most favorable condition for both germination speed and seedling development.

Furthermore, fungal contamination observed at 32 °C may explain the decline in radicle growth at later stages despite initially favorable germination rates. This has been reported in other species, where supra-optimal temperatures accelerate germination but may induce stress, reducing final growth performance (Scott et al., 1984). No germination occurred at 5 °C, indicating that this temperature is below the physiological threshold required for radicle emergence. According to Bewley and Black (2013), temperature regulates germination by influencing the capacity and rate of germination in non-dormant seeds. In this study, seeds germinated rapidly and successfully at 24 °C and 28 °C without any prior cold treatment or stratification protocol, which suggests that *P. vera* seeds, under the specific conditions tested (dehulled seeds soaked for 48 h), do not require a chilling period to achieve successful germination. The absence of germination at constant 5 °C likely reflects a limitation in metabolic activity at this temperature rather than an unfulfilled chilling requirement, since seeds were capable of germinating readily when transferred directly to warm temperatures without any cold pre-treatment.

The satisfactory germination observed in *P. vera* seeds after endocarp removal, water soaking and fungicide treatment is consistent with the findings of Einali and Valizadeh (2017), who reported that pistachio kernels are not deeply dormant. However, it is important to recognize that these results apply specifically to seeds from which the endocarp had been removed prior to germination. Endocarp removal can itself be considered a mechanical scarification treatment, as the hard endocarp is known to physically restrict radicle emergence and limit water uptake in intact seeds. Therefore, the absence of additional dormancy-breaking requirements observed in this study cannot be generalized to intact *P. vera* seeds with the endocarp present. This distinction is relevant when comparing our results with other *Pistacia* species that require stratification or dormancy-breaking methods to achieve successful germination, such as *P. atlantica*, *P. lentiscus*, and *P. terebinthus* (İsfendiyaroğlu and Özeker, 2001; Lefi et al., 2023). Although many studies on *P. vera* report rapid germination without complex treatments, others describe varying degrees of physiological dormancy or even a requirement for cold stratification (Baygi et al., 2015; Fadaei et al., 2009). These contrasting results may be a combination of environmental, physiological and methodological factors that strongly influence the germination of *P. vera* seeds. One key factor is the timing of harvest, seeds collected very late in the season, especially in regions where temperature drops sharply in late autumn, may begin to undergo dormancy induction as a response to early cold exposure or environmental stress (Lachabrouilli et al., 2021).

Pistachio seeds soaked in water for 48 h showed satisfactory and rapid germination under the experimental conditions used in this study, consistent with previous reports describing the positive effect of water soaking on seed hydration and germination performance (Abou Rayya et al., 2018). This characteristic facilitates rapid and uniform seedling emergence, thereby accelerating seedling production and increasing the efficiency of early selection stages in breeding trials (Baygi et al., 2015).

### 4.2 Female parent effects on seed emergence and seedling growth

Although emergence percentage did not differ significantly between the 11 female varieties, consistent trends were observed. Varieties such as ‘Kalehghouchi’, ‘Kerman’, and ‘Golden Hills’ consistently recorded higher emergence rates (>92%), suggesting better seed physiological quality or viability.

In contrast, seedling growth differed significantly among varieties with ‘Kalehghouchi’, ‘Lost Hills’, and ‘Kastel’ producing taller seedlings. This finding suggests that seedling growth potential is influenced by the female variety, although it should be noted that since all seeds were obtained via open pollination, the observed differences cannot be attributed exclusively to maternal genetic effects. Nevertheless, this approach follows established precedent in pistachio breeding research, where open-pollinated half-sib designs have successfully yielded meaningful heritability estimates for key agronomic traits (Chao et al., 1997), supporting the validity of interpreting variety-level differences despite this limitation.

Previous studies have reported significant variation in seedling height and trunk diameter among pistachio genotypes grown under uniform conditions, with differences attributed to genetic polymorphism rather than environmental effects (Oz Barazani et al., 2003) and similar genotype-dependent growth responses have been observed in other tree species (Oyekale et al., 2019).

Interestingly, varieties with higher germination rates did not always correspond to greater seedling vigor, suggesting that emergence and early growth are regulated by partially distinct physiological processes.

### 4.3 Field site effects on seed emergence and seedling growth

The present study revealed varietal differences in both seedling height and seed germination rates between ‘Kerman’ and ‘Lost Hills’, across three distinct field locations. These findings indicate that ‘Lost Hills’ exhibited superior germination capacity and seedling vigor, highlighting the importance of genetic factors in determining early growth performance (Ferguson and Haviland, 2016). However, field site significantly affected only the second seedling height (H2), indicating that the environment in which seeds developed may influence later seedling growth and seed physiological quality. The observed field effect should be interpreted with caution, because seeds were produced under open-pollination conditions, differences among fields may reflect not only variation in environmental conditions during seed development (including climate, soil properties, orchard management, and other site-specific factors), but also differences in paternal genetic composition resulting from the different male pollinator populations present at each orchard.

The marked reduction in germination of ‘Kerman’ seeds produced in the Carmona field illustrates that although varietal rankings remain unchanged, the performance of a seed can be affected by its field origin. Maternal effects were significant at the early growth stage (H1), whereas field effect became more pronounced at the second seedling growth (H2). Overall, these findings suggest that seed origin is an important factor influencing early seedling performance. However, this effect likely represents a combination of environmental influences experienced during seed development and differences in paternal genetic contribution associated with open pollination. Further studies based on controlled crosses or common pollen sources would be required to distinguish between environmental and genetic effects.

### 4.4 Seed weight effect on seed germination and seedling growth

Seed size can significantly influence both germination and seedling growth through several mechanisms. Larger seeds often contain greater reserves of carbohydrates and lipids providing more energy for the initial stage of germination and seedling development (van Mölken et al., 2005; He et al., 2007). In peach, heavier seeds were generally associated with higher germination percentages and more vigorous seedlings, suggesting a positive relationship between seed mass and early seedling development (P.J. Malcolm et al., 2003). However, the relationship is not always consistent. In some cases smaller seeds may germinate faster. For instance, in *Copafeira langsdorffii*, smaller seeds exhibited faster germination and higher germination percentages than larger seeds, although seedlings from larger seeds developed more slowly yet ultimately showed greater vigor (Souza and Fagundes, 2014).

In our study, while seed weight showed a significant correlation with seedling growth, no relationship was observed between seed weight and germination percentage. This finding is consistent with studies in sweet chestnut (*Castanea sativa* Mill.) where seed size positively influenced seedling height but showed no correlation with germination rate (Tumpa et al., 2021). Seed weight is also commonly associated with other morphological and physiological seed traits that may influence imbibition dynamics and reserve mobilization during germination (Pompelli et al., 2023). Nevertheless, in the present work, seed weight was evaluated primarily as a varietal characteristic rather than as an independent experimental factor through predefined seed-weight classes. Therefore, differences in germination speed, uniformity, or imbibition behavior among seed-size categories could not be specifically assessed and should be interpreted cautiously.

The observed variation in seed weight, germination percentage, and seedling vigor across female pistachio cultivars highlights the importance of maternal influence on early developmental traits. Although seedlings from the same maternal genotype were derived from open pollinated seeds, the results suggested that the maternal genotype still applies to a measurable effect on seed and seedling performance, despite the underlying genetic heterogeneity.

The association of certain cultivars with favorable seedling traits, particularly ‘Kalehghouchi’, ‘Lost Hills’, and ‘Kastel’ points to their potential as superior maternal lines in breeding and propagation programs. These varieties may contribute either genetically or physiologically to early seedling vigor, an essential trait for successful orchard establishment and nursery management.

All seeds in this study were obtained through open pollination, and therefore the pollen parent remained unknown. Since pistachio pollen concentration decreases sharply with distance but can still reach female flowers up to 80 m from the source (Erdogan et al., 1997), each progeny may have been fertilized by different male donors present in the experimental fields. Consequently, the observed variation in germination and seedling growth reflects the combined and inseparable influence of both parental genotypes.

This paternal contribution may be relevant, as some xenia effects on kernel weight have been reported in *P. vera* (Crane and Iwakiri, 1980). Nevertheless, the California pistachio improvement program also relied on open-pollinated half-sib families and obtained moderate or even high heritabilities for key agronomic traits (Chao et al., 1997), supporting the validity of this approach. Future studies using controlled crosses would enable a clearer separation of maternal versus paternal contributions.

Overall, the results of this study contribute to the optimization of the early stages of pistachio breeding programs by providing clear criteria for early selection based on germination performance, seedling vigor, and parental effects. The optimal germination temperature for *P. vera* was 28 °C, and 48-hour water soaking was sufficient to achieve rapid germination without dormancy-breaking treatments. The identification of optimal germination temperatures, together with the assessment of maternal, environmental, and seed size influences on early seedling development, enhances the efficiency of seedling production and early-stage evaluation. These findings, although based on open-pollinated progenies, support a reduction in both the time and resources required for the development of new pistachio cultivars, ultimately accelerating the early stages of pistachio breeding.

## Data Availability

The original contributions presented in the study are included in the article/Supplementary Material. Further inquiries can be directed to the corresponding author.
